# Molecular characterisation of phenylketonuria in a Chinese mainland population using next-generation sequencing

**DOI:** 10.1038/srep15769

**Published:** 2015-10-27

**Authors:** Nana Li, Haitao Jia, Zhen Liu, Jing Tao, Song Chen, Xiaohong Li, Ying Deng, Xi Jin, Jiaping Song, Liangtao Zhang, Yu Liang, Wei Wang, Jun Zhu

**Affiliations:** 1 National Center for Birth Defect Monitoring, West China Second University Hospital, Sichuan University, Sec. 3 No. 20, South RenMin Road, Chengdu, Sichuan, China; 2 Key Laboratory of Birth Defects and Related Diseases of Women and Children, Ministry of Education, West China Second University Hospital, Sichuan University, Sec. 3 No. 20, South RenMin Road, Chengdu, Sichuan, China; 3 BGI-Shenzhen, Building No. 11, Beishan Industrial Zone, Yantian District, Shenzhen, Guangdong China; 4 Laboratory of Molecular Epidemiology for birth defect, West China Institute of Women and Children’s Health, Sichuan University, Chengdu, China

## Abstract

Phenylketonuria (PKU) is an inherited autosomal recessive disorder of phenylalanine metabolism, mainly caused by a deficiency of phenylalanine hydroxylase (*PAH*). The incidence of various *PAH* mutations differs among race and ethnicity. Here we report a spectrum of *PAH* mutations complied from 796 PKU patients from mainland China. The all 13 exons and adjacent intronic regions of the *PAH* gene were determined by next-generation sequencing. We identified 194 different mutations, of which 41 are not reported before. Several mutations reoccurred with high frequency including p.R243Q, p.EX6-96A > G, p.V399V, p.R241C, p.R111*, p.Y356*, p.R413P, and IVS4-1G > A. 76.33% of mutations were localized in exons 3, 6, 7, 11, 12. We further compared the frequency of each mutation between populations in northern and southern China, and found significant differences in 19 mutations. Furthermore, we identified 101 mutations that are not reported before in Chinese population, our study thus broadens the mutational spectrum of Chinese PKU patients. Additionally, 41 novel mutations will expand and improve *PAH* mutation database. Finally, our study offers proof that NGS is effective, reduces screening times and costs, and facilitates the provision of appropriate genetic counseling for PKU patients.

Phenylketonuria (PKU, OMIM #261600) is an inherited disorder characterized by increased level of phenylalanine in the blood. PKU is frequently caused by functional deficiency of phenylalanine hydroxylase (*PAH*), an enzyme that converts phenylalanine to other compounds in the body. More than 900 mutations have been identified in *PAH*, and recorded in the locus-specific database (LSD) known as *PAH*vdb (http://www.biopku.org/pah/). The occurrence of PKU varies among ethic and geographic regions, reaching approximately 1 in 15,000 newborns^1^. In mainland China, the average incidence is 1in 11,614^2^, and in Taiwan 1in 55,057^3^.

The frequencies and distributions of *PAH* mutations also differs among different populations^4^,^5^,^6^,^7^,^8^,^9^,^10^,^11^,^12^,^13^,^14^,^15^,^16^,^17^,^18^,^19^,^20^,^21^,^22^,^23^,^24^,^25^,^26^,^27^,^28^,^29^,^30^,^31^,^32^. The wide variability of common mutations between ethnic groups and geographical areas makes *PAH* deficiency a genetic disease of great allelic heterogeneity. Comprehensive catalog of *PAH* mutation spectrum will be of value to evaluate genotype-phenotype correlations, to provide genetic consultations to patients’ families as well as prenatal diagnosis, and to refine diagnoses in and anticipate the dietary requirements of affected patients^33^,^34^,^35^.

A large-scale, unbiased comprehensive survey of *PAH* mutations in Chinese population was not available. Most previous analyses concerning the Chinese population were limited to a few common mutations, or were confined to a certain region of *PAH*, resulting in a selective bias^3^,^12^,^36^,^37^,^38^,^39^,^40^,^41^. One report carried out survey of entire *PAH* in a small-scale survey including 212 patients in mainland China^12^.

In the present study, we report a spectrum of *PAH* mutations complied from a large cohort of 796 PKU patients in mainland China. We determined the sequence of entire *PAH* gene using next-generation sequencing (NGS). Among 194 mutations identified, 41 were not reported in literature, and 101 not reported in Chinese population. We believe that these results will facilitate the development of appropriate genetic counseling for PKU patients in China.

## Results

### Mutation spectrum

In the included cohort of Chinese patients, potential disease-causing mutations were identified on 1516 of 1592 independent alleles, corresponding to a mutation detection rate of 95.23%.A total of 720 patients were completely genotyped, whereas in the remaining 76 individuals only one causative mutation was identified; the other mutation site could not be identified by this platform. Among the fully genotyped patients, two mutations were detected in 683 of the patients, who had either compound heterozygous (n = 622) or homozygous (n = 61) genotypes, three mutations were found in 35 of the patients, and four mutations were revealed in 2 of the patients. The gene analysis results were summarised in Table 1.

A total of 194 different types of mutations were identified, including 134 missense mutations (69.07%), 25 splice-site mutations (12.89%), 18 deletions (9.28%), 14 nonsense mutations (7.22%), 2 insertions (1.03%), and 1 silence/splice (0.52%). 76.33% of the total mutations are found in exons. Most mutations was localized in exon7 (33.44%), exon11 (13.18%), exon6 (10.48%), exon12 (10.29%), exon3 (8.94%). Interestingly, no mutations were identified in exon13.

In terms of mutation frequency, p.R243Q was the most prevalent mutation (frequency 17.53%). Other mutations with relatively high frequencies were p.EX6-96A > G, p.V399V, p.R241C, p.R111*, p.Y356*, p.R413P, and IVS4-1G > A (7.66%, 5.84%, 5.40%, 4.77%, 4.46%, 4.33%, 3.77%, respectively). Eight mutations, including p.R53H, p.A434D, p.R408Q, IVS7 + 2T > A, p.G247V, p.S70del, p.R261Q, and p.Y166* were found with relatively lower frequencies (ranging from 1% to 3%). The remaining 178 mutations were found at relative frequencies of less than 1%.

### Comparison between northern and southern Chinese populations

The frequencies of each *PAH* mutations were compared among geographical regions in China. We used sixteen mutations with frequency more than 1% for this comparison. Six mutations showed obvious local mutation clustering: p.V399V, p.R413P and IVS7 + 2T > A were found to be clustered in northern Chinese populations, and p.R241C, p.R408Q and p.Y166* were clustered in southern Chinese populations. Among the remaining 178 mutations that exhibited a relative frequency of <1%, thirteen exhibited significant differential distribution in northern and southern China.

### Novel sequence variants

Forty-one novel nucleotide lesions that have not been registered in the BIOPKU database (http://www.biopku.org/pah/) were identified in this study: p.V5Sfs*34, IVS1 + 5_ + 6delGC, IVS1-3T > C, p.F39del, p.E43*, p.N58*, p.L62Pfs*7, p.E78Q, p.E78V, p.I95del, p.D101N, p.G103D, IVS3-2A > G, p.F121V, p.E127K, p.E127G, p.G148R, p.R169S, p.R169G, p.H170P, p.E178K, p.N223I, p.F260I, p.E280Nfs*61, p.V291M, p.V291L, p.P292S, p.D296H, p.Q304K, p.T328N, p.K335E, p.A342Hfs*59, p.Y343*, IVS10-12delT, IVS10-1G > C, IVS10-1G > T, p.Y356D, IVS11-1G > C, p.T372R, p.I406V, p.Q429K.

Among these mutations, the majority were missense mutations (n = 25), followed by small deletions (n = 8), nonsense (n = 2), splice (n = 5) and insertions (n = 1). Thirty-four of the novel mutations were detected in coding regions, and the remaining were located in introns.

The prediction results of novel mutations are listed in Table 2. A total of 25 novel mutations were predicted; of these, 20 mutations were predicted to be probably damaging, 5 mutations were tolerated, and the remainder of the 16 mutations could not be predicted using this tool. The frequencies of the novel mutations were relatively low, which indicates that they are rare mutations.

## Discussion

A comprehensive survey of the mutation spectrum of the protein of interest in a given population not only can provide insight into the structural and functional aspects of the protein as well as genotype-phenotype correlations^14^,^15^,^20^,^22^,^23^,^26^,^28^,^42^, but also facilitate genetic counseling in patients’ families. In this study, we described the molecular basis of PKU in a mainland Chinese population by analysing mutations in the *PAH* gene using NGS. Among a cohort of 796 patients, mutations were detected on 1516 of the 1592 independent alleles, representing a mutation detection rate of 95.23% (Table 1). A total of 194 distinct mutations were found, demonstrating the high genetic heterogeneity that is inherent in PKU.

The number of different mutations in a given population is usually high, and is typically comprised of a few prevalent mutations and a large number of private mutations^43^. In comparing our study with previous reports, 83 of the mutations that we identified have been previously reported; however, the remaining 101 mutations (including 41 novel mutations) were reported for the first time in a Chinese mainland population^12^,^36^,^37^,^38^,^39^,^41^. As shown in Table 3, eight mutations including p.R243Q, p.EX6-96A > G, p.V399V, p.R241C, p.R111*, p.Y356*, p.R413P and IVS4-1G > A are common mutations in the mainland Chinese population, although the rank order of these mutations was different. Among them, R241C, p.EX6-96A > G, p.V399V, R413P, R243Q and R111* are also considered to be prevalent in the Chinese Taiwanese population^40^. The epidemiology of phenylketonuria in China is complicated. The prevalence of PKU in northern China (1/11,000) is close to what has been documented in Caucasian populations, but the prevalence in southern China is much lower^44^. A comparison between northern and southern China indicated marked differences in the relative frequencies of mutations. Among eight common mutations, V399V, R241C, and R413P gave significant p values between two regions, with respective p values of 0.037, 0.007, and 0.002. The result that p.R413P clustered in northern China is consistent with what has been reported by Gu. *et al.*^12^. Furthermore, p.V399V has been previously detected primarily in populations of Xinjiang and northern China^36^,^37^, whereas p.R241C has been primarily detected in populations in southern China and in Taiwan patients^3^,^45^. The majority of the population in Taiwan has descended from southeast China. Based on our data, we hypothesize that the uniform distribution of V399V, R241C, and R413P is a result of migration and the founder effect.

It is well known that different ethnic groups have their own distinctive and diverse *PAH* mutant allele series that include either one or a few prevalent founder alleles^46^. When comparing *PAH* mutational data between different ethnic groups, correlations between the mutations in and the genetic histories of the investigated populations were found. Marked differences were identified when comparing *PAH* mutations with ≥ 3% frequency (totaling 34) between Asian and European countries (Table 4). Five mutations, including p.R243Q, p.EX6-96A > G, p.R241C, p.R413P and IVS4-1G > A, were found to be common mutations in East Asian countries such as China, Japan^14^ and Korea^8^, accounting for 53.76%, 69.70%, and 62.10% of the total mutations respectively. Three mutations, including p.R111*, p.Y356* and p.T278I, were frequently detected in China and Japan^14^, China and Korea^8^, Japan^14^ and Korea^8^ respectively. The remaining mutations, including p.V399V, p.A259T, p.R252W, p.Y325* and p.V388M, were found to be common in only one country. In sharp contrast, these mutations except for p.R252W, the above mentioned mutations were either rarely detected or undetected in West Asia and Europe countries. For example in Iran^17^ and Turkey^18^, three common mutations including p.R261Q, p.P281L, and IVS10-11G, A were shared, However these mutations were either rare or did not occur in East Asia. Instead, they were prevalent in select Europe countries. In Europe, p.R408W was found to be the common mutation, ranking first in the Czech Republic^28^ (East Europe) and Germany^5^ (West Europe), and second in Danemark^4^ (North Europe) and Serbia^32^(South Europe). These results suggest that p.R408W was the most prevalent founder allele in the European population^46^. In contrast, p.R408W was either rarely detected or undetected in populations of East Asia, whereas it was the most common mutation in Turkish populations^18^. The remaining mutations were only found to be prevalent in subsets of the four countries. For example, p.L48S was the most prevalent mutation in Serbian populations^32^, and it was also common in Turkish populations^18^. The p.R158Q, p.A403V, p.Y414C, and IVS12 + 1G > A mutations were relatively common in only two countries. Based on the above comparison, we identified that there were several overlaps of mutant allele distributions between West Asia and Europe and that the mutations that were common in East Asia were different from these.

In the present study, the high mutation detection rate of 95.23% was similar to previous studies in which sequencing analysis of the *PAH* gene was conducted^12^,^15^, but it was relatively lower than the results from studies that employed exon analysis combined with multiplex ligation-dependent probe amplification (MLPA)^14^,^20^,^24^,^28^. This is because NGS is able to detect small deletions and insertions, whereas it is not able to detect large deletions or duplications. Despite scanning the entirety of the *PAH* coding region and its exon–intron boundaries, no mutations were detected in 76 alleles. The most likely explanation behind this is that the mutations are located in regions that were not detectable in this study (for example, in the promoter regions, the 5′ and 3′ UTRs, in non-coding RNA binding sites, or in the intronic sequences far away from exon–intron boundaries). Alternatively, the mutations may have been large deletions or duplications.

Using NGS as a routine genetic diagnostic tool enables thousands of DNA sequences to be simultaneously obtained in notably reduced turnaround times and at a significantly reduced cost. Furthermore, this technique provides high sensitivity, specificity and coverage (including all coding regions of the involved exons and adjacent intronic regions). However, the biggest limitation to using NGS is the need to analyse and interpret complicated data.

We believe our study will provide guidance for future medical practice such as prenatal screening and early diagnosis of PKU. Diagnostic methods can be developed based on the known characteristics of a population. Currently, PKU screening is performed in newborn babies as a part of the tertiary prevention in birth defects preventive network in China. Developing a new method for screening might enhance primary prevention. Based on the mutational spectrum presented in this study, our hope is that carrier screening can be conducted preceding gestation, which would offer timely guidance with respect to prenatal diagnosis for couples who are both carriers.

## Methods

### Subjects

A total of 796 unrelated patients from 29 separate newborn screening centres of China were enrolled. These patients were diagnosed at birth either through a neonatal screening program or based on clinical presentation. Demographic data, including age, consanguinity, family history, and geographical origin, and biochemical testing data, including plasma phenylalanine (Phe) levels, dihydropteridine reductase activity, urinary biopterin and neopterin ratio, and tetrahydrobiopterin loading, were collected. The ages of the included patients ranged from 6- months to 5-years old. In families with more than one patient, only one member of each sibling pair was included in the study of mutation frequency. The numbers of patients in northern China and southern China that were divided by the Qinling Mountains and the Huaihe River were 557 and 239, respectively. Both parents of the included patients were native.

These patients were classified into one of three separate phenotype categories according to their pretreatment plasma Phe levels, including mild hyperphenylalaninaemia (Phe 120–600 umol/L), mild PKU(Phe 600-1200 μmol/L), and classic PKU (Phe >1200 μmol/L)^47^. Among the 796 patients, 145 (18.22%) were mild hyperphenylalaninemia(MHP), 215 (27.01%) were mild PKU(mPKU), and 418 (52.51%) were classic PKU(cPKU), the remaining 18 patients (2.26%) could not be classified because the pretreatment Phe levels were not available(Table 5). The entire list of patients’ phenotype and blood Phe levels can be found as Supplementary Table S1. The parental permissions and informed consents were obtained from the parents of all patients. The research was approved by the Ethics Committee of West China Second University Hospital, Sichuan University (No: 2014018) and followed the tenets of the Declaration of Helsinki.

### DNA extraction

Blood samples (4 ml) were collected from each patient and their parents by venipuncture in EDTA. Genomic DNA was extracted from peripheral blood leukocytes using a QIAamp® DNA Blood Mini Kit (Qiagen, Cat.No.51106, Germany) according to the recommended protocol.

### Next-generation sequencing

A series of unique primers were designed to amplify all 13 exons and their surrounding introns, which covered 200bp upstream and 200bp downstream of the exons of the *PAH* gene. A pair of primers that were designed to amplify approximately 150 bp of the human β-globin gene (*HBB)* (accession number AY260740) was used as an internal quality control to identify false negatives caused by inadequate DNA or failed PCR.

To improve the throughput of the assay, the PCR primers were designed to not only amplify the target DNA but also to provide a unique primer index for each of the 96 samples in each of the plates (multiple index PCR). This strategy resulted in 96 sets of 10-bp-long nucleic acid tags that were individually included at the 5′ ends of each of the *PAH* and *HBB* primers. The second index that was used for each sample was the 8-bp-long nucleic acid tag from the library adapter sequence, which identified the specific 96-well plate that each sample was included in. This index was attached to the amplicons of the samples through an adapter preparation process. Using this “double index system,” hundreds of samples can be mixed together and detected in one sequencing chip at the same time.

PCR was performed on a GeneAmp PCR system 9700 (Applied Biosystems, Foster City, CA), with a cycling protocol that consisted of denaturation at 94 °C for 30 s, 56° C for 30 s, and 72 °C for 1 min. After 35 cycles, gel electrophoresis was used to verify the quality of the amplified DNA, only eligible DNAs was included in the library preparation. The PCR amplification products were prepared for DNA pooling, and the diverse adapter library was added onto the amplification products. After a concentration of DNA was obtained that could satisfy the requirements of the library preparation method, gene mutations were sequenced using an Illumina Hiseq 2000 (Illumina Inc, San Diego, CA, USA) sequencing instrument.

After sequencing the samples, the raw sequence data were analysed using in-house software. First, all sequence data were traced back to the specimens from which they arose according to the sequences of the primer and adapter indices. Second, the amplicon sequences of each of the samples were aligned with standard reference *PAH* sequences from the database *PAH*vdb (http://www.biopku.org/pah/); SNPs were found in target areas and relevant information was noted.

All of the *PAH* gene sequencing reactions and analyses were performed in the Centre of BGI Health clinical laboratory, Shenzhen, China.

### Validation tests of Sanger sequencing

When a given patient’s mutation locus was detected, it was amplified by polymerase chain reaction (PCR) from a parent’s sample and then sequenced bidirectionally in an ABI-3730 DNA analyser. This not only validated the locus but also confirmed the carrier status of the parents. The PCR cycling protocol consisted of an initial denaturation at 95 °C for 3 min, followed by 35 cycles, of 95 °C for 40 s, 55 °C for 30 s, and 72 °C for 30 s, with a final extension at 72 °C for 10 min.

### Pathologic analysis of novel mutations

A SIFT prediction was performed (http://sift.jcvi.org/) using the “SIFT Human SNPs” tool to obtain predictions for nonsynonymous SNPs. The annotation version was Homo sapiens GRCh37 Ensembl 63. A list of the chromosomal positions and alleles corresponding to the 41 novel mutations were uploaded into the import the web-site.

### Statistical analysis

Statistical analysis was performed using statistical package for social science software (SPSS version 16.0). Mutational frequencies were calculated by the counting method. An x^2^ analysis was performed to test for differences between two geographic populations. A p value <0.05 was considered statistically significant.

## Additional Information

**How to cite this article**: Li, N. *et al.* Molecular characterisation of phenylketonuria in a Chinese mainland population using next-generation sequencing. *Sci. Rep.* **5**, 15769; doi: 10.1038/srep15769 (2015).

## Supplementary Material

Supplementary Information

## Figures and Tables

**Table 1:** The spectrum of *PAH* mutations in Chinese Mainland PKU patients.

Trivial name (protein effect)	Syetematic name (DNA level)	Location	Characters of mutation	No. of alleles	RF(%)	No. of alleles	RF(%)	No. of alleles	RF(%)	p value
				Chinese (n = 796)		Northern China (n = 557)		Southern China (n = 239)		
p.R243Q	c.728G > A	E7	Missense	279	17.53	202	18.13	77	16.11	0.3300
p.EX6-96A > G	c.611A > G	E6	Splice	122	7.66	84	7.54	38	7.95	0.7780
p.V399V	c.1197A > T	E11	Silence/Splice	93	5.84	74	6.64	19	3.97	0.0370**
p.R241C	c.721C > T	E7	Missense	86	5.40	49	4.40	37	7.74	0.0070**
p.R111*	c.331C > T	E3	Nonsense	76	4.77	60	5.39	16	3.35	0.0800
p.Y356*	c.1068C > A	E11	Nonsense	71	4.46	57	5.12	14	2.93	0.0530
p.R413P	c.1238G > C	E12	Missense	69	4.33	60	5.39	9	1.88	0.0020**
IVS4-1G > A	c.442-1G > A	I4	Splice	60	3.77	36	3.23	24	5.02	0.0860
p.R53H	c.158G > A	E2	Missense	40	2.51	33	2.96	7	1.46	0.0800
p.A434D	c.1301C > A	E12	Missense	29	1.82	19	1.71	10	2.09	0.5970
p.R408Q	c.1223G > A	E12	Missense	26	1.63	13	1.17	13	2.72	0.0250**
IVS7 + 2T > A	c.842 + 2T > A	I7	Splice	24	1.51	22	1.97	2	0.42	0.0190**
p.G247V	c.740G > T	E7	Missense	23	1.44	20	1.80	3	0.63	0.0730
p.S70del	c.206_208delCTT	E3	Deletion	20	1.26	13	1.17	7	1.46	0.6250
p.R261Q	c.782G > A	E7	Missense	20	1.26	12	1.07	8	1.67	0.3270
p.Y166*	c.498C > G	E5	Nonsense	17	1.07	8	0.72	9	1.88	0.0380**
p.R241Pfs*100	c.722delG	E7	Deletion	14	0.88	3	0.27	3	0.63	0.2850
p.F161S	c.482T > C	E5	Missense	13	0.82	11	0.99	2	0.42	0.2480
p.R176*	c.526C > T	E6	Nonsense	13	0.82	9	0.81	4	0.84	0.9530
p.G247R	c.739G > C	E7	Missense	13	0.82	10	0.90	3	0.63	0.5830
p.S349A	c.1045T > G	E10	Missense	12	0.75	10	0.90	2	0.42	0.3110
p.I65T	c.194T > C	E3	Missense	12	0.75	9	0.81	3	0.63	0.7030
p.R252Q	c.755G > A	E7	Missense	12	0.75	6	0.54	6	1.26	0.1300
p.G257V	c.770G > T	E7	Missense	11	0.69	4	0.36	7	1.46	0.0150**
p.Q419R	c.1256A > G	E12	Missense	9	0.57	9	0.81	0	0.00	0.0490**
p.Y325*	c.975C > G	E10	Nonsense	9	0.57	5	0.45	4	0.84	0.3440
p.A345T	c.1033G > A	E10	Missense	8	0.50	5	0.45	3	0.63	0.6440
p.R408W	c.1222C > T	E12	Missense	8	0.50	6	0.54	2	0.42	0.7560
IVS12 + 6T > A	c.1315 + 6T > A	I12	Splice	8	0.50	5	0.45	3	0.63	0.6440
p.E56D	c.168G > T	E2	Missense	8	0.50	8	0.72	0	0.00	0.0630
p.S70del	c.208_210delTCT	E3	Deletion	8	0.50	5	0.45	3	0.63	0.6440
IVS4 + 3G > C	c.441 + 3G > C	I4	Splice	8	0.50	7	0.63	1	0.21	0.2780
p.A156P	c.466G > C	E5	Missense	8	0.50	4	0.36	4	0.84	0.2170
p.R158Q	c.473G > A	E5	Missense	8	0.50	7	0.63	1	0.21	0.2780
p.M276K	c.827T > A	E7	Missense	8	0.50	7	0.63	1	0.21	0.2780
p.E280K	c.838G > A	E7	Missense	8	0.50	7	0.63	1	0.21	0.2780
p.V388M	c.1162G > A	E11	Missense	7	0.44	2	0.18	5	1.05	0.0170**
p.R400T	c.1199G > C	E11	Missense	7	0.44	4	0.36	3	0.63	0.4580
p.R158W	c.472C > T	E5	Missense	7	0.44	5	0.45	2	0.42	0.9330
p.L255S	c.764T > C	E7	Missense	7	0.44	6	0.54	1	0.21	0.3630
p.I324N	c.971T > A	E10	Missense	7	0.44	7	0.63	0	0.00	0.0820
p.F392I	c.1174T > A	E11	Missense	6	0.38	3	0.27	3	0.63	0.2850
p.R400K	c.1199G > A	E11	Missense	6	0.38	6	0.54	0	0.00	0.1080
p.T418P	c.1252A > C	E12	Missense	6	0.38	3	0.27	3	0.63	0.2850
p.Q232*	c.694C > T	E6	Missense	6	0.38	5	0.45	1	0.21	0.4740
IVS6-1G > A	c.707-1G > A	I6	Splice	6	0.38	4	0.36	2	0.42	0.8590
p.R241H	c.722G > A	E7	Missense	6	0.38	4	0.36	10	2.09	0.0010**
p.W326*	c.977G > A	E10	Nonsense	6	0.38	4	0.36	2	0.42	0.8590
IVS10-11G > A	c.1066-11G > A	I10	Splice	4	0.25	3	0.27	1	0.21	0.8260
p.H107R	c.320A > G	E3	Missense	4	0.25	2	0.18	2	0.42	0.3830
p.R252W	c.754C > T	E7	Missense	4	0.25	1	0.09	3	0.63	0.0490**
p.R261*	c.781C > T	E7	Nonsense	4	0.25	3	0.27	1	0.21	0.8260
IVS7-14_-11delCTTT	c.843-14_-11delCTTT	I7	Deletion	4	0.25	3	0.27	1	0.21	0.8260
IVS8-7A > G	c.913-7A > G	I8	Splice	4	0.25	3	0.27	1	0.21	0.8260
p.A342Hfs*59#	c.1023delG	E10	Deletion	3	0.19	2	0.18	1	0.21	0.9000
p.F39del #	c.113_115delTCT	E2	Deletion	3	0.19	2	0.18	1	0.21	0.9000
IVS11-1G > C#	c.1200-1G > C	I11	Splice	3	0.19	0	0.00	3	0.63	0.0080
p.I421T	c.1262T > C	E12	Missense	3	0.19	3	0.27	0	0.00	0.2560
IVS12 + 4A > G	c.1315 + 4A > G	I12	Splice	3	0.19	1	0.09	2	0.42	0.1660
IVS12-2A > C	c.1316-2A > C	I12	Splice	3	0.19	1	0.09	2	0.42	0.1660
p.A47E	c.140C > A	E2	Missense	3	0.19	0	0.00	3	0.63	0.0080**
IVS2 + 5G > C	c.168 + 5G > C	I2	Splice	3	0.19	3	0.27	0	0.00	0.2560
p.L98V	c.292T > G	E3	Missense	3	0.19	2	0.18	1	0.21	0.9000
p.V230I	c.688G > A	E6	Missense	3	0.19	3	0.27	0	0.00	0.2560
p.F233L	c.699C > G	E6	Missense	3	0.19	2	0.18	1	0.21	0.9000
p.T278I	c.833C > T	E7	Missense	3	0.19	3	0.27	0	0.00	0.2560
p.P281L	c.842C > T	E7	Missense	3	0.19	2	0.18	1	0.21	0.9000
p.F331S	c.992T > C	E10	Missense	3	0.19	1	0.09	2	0.42	0.1660
p.A342T	c.1024G > A	E10	Missense	2	0.13	2	0.18	0	0.00	0.3540
p.S350Y	c.1049C > A	E10	Missense	2	0.13	2	0.18	0	0.00	0.3540
IVS10-1G > A	c.1066-1G > A	I10	Splice	2	0.13	2	0.18	0	0.00	0.3540
p.S359L	c.1076C > T	E11	Missense	2	0.13	2	0.18	0	0.00	0.3540
p.K363Nfs*37	c.1089delG	E11	Deletion	2	0.13	2	0.18	0	0.00	0.3540
p.T372R#	c.1115C > G	E11	Missense	2	0.13	2	0.18	0	0.00	0.3540
p.Y387D	c.1159T > G	E11	Missense	2	0.13	2	0.18	0	0.00	0.3540
p.A403V	c.1208C > T	E12	Missense	2	0.13	1	0.09	1	0.21	0.5370
p.I406Sfs*17	c. 1215_1219delAATAC	E12	Deletion	2	0.13	1	0.09	1	0.21	0.5370
p.D415N	c.1243G > A	E12	Missense	2	0.13	1	0.09	1	0.21	0.5370
p.E43*#	c.127G > T	E2	Nonsense	2	0.13	2	0.18	0	0.00	0.3540
p.R71H	c.212G > A	E3	Missense	2	0.13	0	0.00	2	0.42	0.0310**
p.I95del #	c.279_281delCAT	E3	Deletion	2	0.13	2	0.18	0	0.00	0.3540
p.G103D#	c.308G > A	E3	Missense	2	0.13	0	0.00	2	0.42	0.0310**
IVS3-2A > T	c.353-2A > T	I3	Splice	2	0.13	1	0.09	1	0.21	0.5370
p.S16*	c.44_45delTC	E1	Deletion	2	0.13	2	0.18	0	0.00	0.3540
p.P147L	c.440C > T	E4	Missense	2	0.13	1	0.09	1	0.21	0.5370
p.G148V	c.443G > T	E5	Missense	2	0.13	2	0.18	0	0.00	0.3540
p.Y154H	c.460T > C	E5	Missense	2	0.13	2	0.18	0	0.00	0.3540
p.A165D	c.494C > A	E5	Missense	2	0.13	2	0.18	0	0.00	0.3540
p.R169G#	c.505C > G	E5	Missense	2	0.13	2	0.18	0	0.00	0.3540
IVS5 + 1G > A	c.509 + 1G > A	I5	Splice	2	0.13	1	0.09	1	0.21	0.5370
p.Y206C	c.617A > G	E6	Missense	2	0.13	2	0.18	0	0.00	0.3540
p.C217Y	c.650G > A	E6	Missense	2	0.13	2	0.18	0	0.00	0.3540
p.H220P	c.659A > C	E6	Missense	2	0.13	2	0.18	0	0.00	0.3540
p.T238A	c.712A > G	E7	Missense	2	0.13	2	0.18	0	0.00	0.3540
p.G239D	c.716G > A	E7	Missense	2	0.13	0	0.00	2	0.42	0.0310**
p.T278A	c.832A > G	E7	Missense	2	0.13	0	0.00	2	0.42	0.0310**
p.S303Pfs*38	c.907delT	E8	Deletion	2	0.13	1	0.09	1	0.21	0.5370
IVS8-2A > G	c.913-2A > G	I8	Splice	2	0.13	0	0.00	2	0.42	0.0310**
p.L308F	c.922C > T	E9	Missense	2	0.13	1	0.09	1	0.21	0.5370
p.G312V	c.935G > T	E9	Missense	2	0.13	0	0.00	2	0.42	0.0310**
p.P314T	c.940C > A	E9	Missense	2	0.13	2	0.18	0	0.00	0.3540
p.K335E#	c.1003A > G	E10	Missense	1	0.06	1	0.09	0	0.00	0.5120
p.Y343*#	c.1029T > A	E10	Nonsense	1	0.06	1	0.09	0	0.00	0.5120
p.G344S	c.1030G > A	E10	Missense	1	0.06	1	0.09	0	0.00	0.5120
p.G344D	c.1031G > A	E10	Missense	1	0.06	1	0.09	0	0.00	0.5120
IVS10 + 1G > T	c.1065 + 1G > T	I10	Splice	1	0.06	1	0.09	0	0.00	0.5120
IVS10-12delT#	c.1066-12delT	I10	Deletion	1	0.06	0	0.00	1	0.21	0.1270
IVS10-1G > C#	c.1066-1G > C	I10	Splice	1	0.06	0	0.00	1	0.21	0.1270
IVS10-1G > T#	c.1066-1G > T	I10	Splice	1	0.06	0	0.00	1	0.21	0.1270
IVS10-3C > T	c.1066-3C > T	I10	Splice	1	0.06	0	0.00	1	0.21	0.1270
p.Y356D#	c.1066T > G	E11	Missense	1	0.06	0	0.00	1	0.21	0.1270
p.C357Y	c.1070G > A	E11	Missense	1	0.06	0	0.00	1	0.21	0.1270
p.P362L	c.1085C > T	E11	Missense	1	0.06	1	0.09	0	0.00	0.5120
p.L367V	c.1099C > G	E11	Missense	1	0.06	0	0.00	1	0.21	0.1270
p.T372S	c.1114A > T	E11	Missense	1	0.06	0	0.00	1	0.21	0.1270
p.Q375E	c.1123C > G	E11	Missense	1	0.06	1	0.09	0	0.00	0.5120
p.F39del	c.116_118delTCT	E11	Deletion	1	0.06	0	0.00	1	0.21	0.1270
IVS11 + 1G > C	c.1199 + 1G > C	I11	Splice	1	0.06	1	0.09	0	0.00	0.5120
p.I406V#	c.1216A > G	E12	Missense	1	0.06	1	0.09	0	0.00	0.5120
p.L41F	c.121C > T	E2	Missense	1	0.06	1	0.09	0	0.00	0.5120
p.D415Y	c.1243G > T	E12	Missense	1	0.06	1	0.09	0	0.00	0.5120
p.Q429K#	c.1285C > A	E12	Missense	1	0.06	1	0.09	0	0.00	0.5120
p.L430P	c.1289T > C	E12	Missense	1	0.06	0	0.00	1	0.21	0.1270
p.V5Sfs*34#	c.13delG	E1	Deletion	1	0.06	0	0.00	1	0.21	0.1270
p.L48S	c.143T > C	E2	Missense	1	0.06	1	0.09	0	0.00	0.5120
p.L52S	c.155T > C	E2	Missense	1	0.06	1	0.09	0	0.00	0.5120
p.N58*#	c.169_170delGA	E3	Deletion	1	0.06	1	0.09	0	0.00	0.5120
p.L62Pfs*7#	c.184_187insCTGA	E3	Insertion	1	0.06	1	0.09	0	0.00	0.5120
p.I65V	c.193A > G	E3	Missense	1	0.06	1	0.09	0	0.00	0.5120
p.I65S	c.194T > G	E3	Missense	1	0.06	1	0.09	0	0.00	0.5120
p.P69S	c.205C > T	E3	Missense	1	0.06	0	0.00	1	0.21	0.1270
p.D75G	c.224A > G	E3	Missense	1	0.06	0	0.00	1	0.21	0.1270
p.D75V	c.224A > T	E3	Missense	1	0.06	1	0.09	0	0.00	0.5120
p.E78Q#	c.232G > C	E3	Missense	1	0.06	0	0.00	1	0.21	0.1270
p.E78V#	c.233A > T	E3	Missense	1	0.06	0	0.00	1	0.21	0.1270
p.D101N#	c.301G > A	E3	Missense	1	0.06	1	0.09	0	0.00	0.5120
p.R13Qfs*5	c.30_31insC	E1	Insertion	1	0.06	1	0.09	0	0.00	0.5120
IVS3-2A > G#	c.353-2A > G	I3	Splice	1	0.06	0	0.00	1	0.21	0.1270
p.F121V#	c.361T > G	E4	Missense	1	0.06	1	0.09	0	0.00	0.5120
p.E127K#	c.379G > A	E4	Missense	1	0.06	0	0.00	1	0.21	0.1270
p.E127G#	c.380A > G	E4	Missense	1	0.06	0	0.00	1	0.21	0.1270
IVS4 + 1G > A	c.441 + 1G > A	I4	Splice	1	0.06	1	0.09	0	0.00	0.5120
IVS4 + 2T > A	c.441 + 2T > A	I4	Splice	1	0.06	1	0.09	0	0.00	0.5120
p.G148R#	c.442G > C	E5	Missense	1	0.06	0	0.00	1	0.21	0.1270
p.R155H	c.464G > A	E5	Missense	1	0.06	0	0.00	1	0.21	0.1270
p.S16*	c.47_48delCT	E1	Deletion	1	0.06	1	0.09	0	0.00	0.5120
p.R157K	c.470G > A	E5	Missense	1	0.06	1	0.09	0	0.00	0.5120
p.Q160*	c.478C > T	E5	Nonsense	1	0.06	0	0.00	1	0.21	0.1270
p.R169S#	c.505C > A	E5	Missense	1	0.06	1	0.09	0	0.00	0.5120
p.R169C	c.505C > T	E5	Missense	1	0.06	1	0.09	0	0.00	0.5120
p.H170P#	c.509A > C	E5	Missense	1	0.06	1	0.09	0	0.00	0.5120
p.H170R	c.509A > G	E5	Missense	1	0.06	1	0.09	0	0.00	0.5120
IVS5-2A > G	c.510-2A > G	I5	Splice	1	0.06	0	0.00	1	0.21	0.1270
p.H170Q	c.510T > A	E6	Missense	1	0.06	1	0.09	0	0.00	0.5120
p.G171A	c.512G > C	E6	Missense	1	0.06	1	0.09	0	0.00	0.5120
p.Q172H	c.516G > T	E6	Missense	1	0.06	0	0.00	1	0.21	0.1270
p.P175S	c.523C > T	E6	Missense	1	0.06	1	0.09	0	0.00	0.5120
p.E178K#	c.532G > A	E6	Missense	1	0.06	1	0.09	0	0.00	0.5120
p.G188D	c.563G > A	E6	Missense	1	0.06	1	0.09	0	0.00	0.5120
p.Q20P	c.59A > C	E1	Missense	1	0.06	0	0.00	1	0.21	0.1270
IVS1 + 5_ + 6delGC#	c.60 + 5_ + 6delGC	I1	Deletion	1	0.06	0	0.00	1	0.21	0.1270
p.Q20H	c.60G > C	E1	Missense	1	0.06	0	0.00	1	0.21	0.1270
IVS1-3T > C#	c.61-3T > C	I1	Splice	1	0.06	1	0.09	0	0.00	0.5120
p.Y206*	c.618C > A	E6	Nonsense	1	0.06	0	0.00	1	0.21	0.1270
p.N223I#	c.668A > T	E6	Missense	1	0.06	1	0.09	0	0.00	0.5120
p.L227Q	c.680T > A	E6	Missense	1	0.06	1	0.09	0	0.00	0.5120
p.S231P	c.691T > C	E6	Missense	1	0.06	1	0.09	0	0.00	0.5120
p.G239A	c.716G > C	E7	Missense	1	0.06	1	0.09	0	0.00	0.5120
p.L242F	c.724C > T	E7	Missense	1	0.06	1	0.09	0	0.00	0.5120
p.R243*	c.727C > T	E7	Nonsense	1	0.06	1	0.09	0	0.00	0.5120
p.L255V	c.763T > G	E7	Missense	1	0.06	1	0.09	0	0.00	0.5120
p.F260I#	c.778T > A	E7	Missense	1	0.06	1	0.09	0	0.00	0.5120
p.C265*	c.795C > A	E7	Nonsense	1	0.06	0	0.00	1	0.21	0.1270
p.Q267*	c.799C > T	E7	Nonsense	1	0.06	1	0.09	0	0.00	0.5120
p.Q267L	c.800A > T	E7	Missense	1	0.06	1	0.09	0	0.00	0.5120
p.H271R	c.812A > G	E7	Missense	1	0.06	1	0.09	0	0.00	0.5120
p.H271L	c.812A > T	E7	Missense	1	0.06	1	0.09	0	0.00	0.5120
p.M276R	c.827T > G	E7	Missense	1	0.06	1	0.09	0	0.00	0.5120
p.E280Nfs*61#	c.833delC	E7	Deletion	1	0.06	0	0.00	1	0.21	0.1270
p.E280G	c.839A > G	E7	Missense	1	0.06	0	0.00	1	0.21	0.1270
IVS7 + 1G > A	c.842 + 1G > A	I7	Splice	1	0.06	0	0.00	1	0.21	0.1270
p.D282G	c.845A > G	E8	Missense	1	0.06	1	0.09	0	0.00	0.5120
p.H285Y	c.853C > T	E8	Missense	1	0.06	0	0.00	1	0.21	0.1270
p.V291M#	c.871G > A	E8	Missense	1	0.06	0	0.00	1	0.21	0.1270
p.V291L#	c.871G > T	E8	Missense	1	0.06	0	0.00	1	0.21	0.1270
p.P292S#	c.874C > T	E8	Missense	1	0.06	1	0.09	0	0.00	0.5120
p.P292L	c.875C > T	E8	Missense	1	0.06	0	0.00	1	0.21	0.1270
p.D296H#	c.886G > C	E8	Missense	1	0.06	1	0.09	0	0.00	0.5120
p.Q304K#	c.910C > A	E8	Missense	1	0.06	1	0.09	0	0.00	0.5120
p.G307D	c.920G > A	E9	Missense	1	0.06	1	0.09	0	0.00	0.5120
p.S310F	c.929C > T	E9	Missense	1	0.06	1	0.09	0	0.00	0.5120
p.A322T	c.964G > A	E9	Missense	1	0.06	1	0.09	0	0.00	0.5120
p.A322D	c.965C > A	E9	Missense	1	0.06	0	0.00	1	0.21	0.1270
p.T328N#	c.983C > A	E10	Missense	1	0.06	0	0.00	1	0.21	0.1270

Abbreviation: *PAH*, phenylalanine hydroxylase; RF, relative frequency; ^#^novel mutations; **p <0.05, p value is for the comparision of RF between Northern China and Southern China groups.

**Table 2:** Pathologic analysis of 25 novel mutations of the *PAH* gene.

Trivial name (protein effect)	Syetematic name (DNA level)	Characters of mutation	SIFT(score)	Trivial name (protein effect)	Syetematic name (DNA level)	Characters of mutation	SIFT(score)
p.E78Q	c.232G > C	Missense	Damaging(0.007)	p.F260I	c.778T > A	Missense	Damaging(0.000)
p.E78V	c.233A > T	Missense	Damaging(0.002)	p.V291M	c.871G > A	Missense	Tolerated(0.209)
p.D101N	c.301G > A	Missense	Tolerated(0.322)	p.V291L	c.871G > T	Missense	Damaging(0.004)
p.G103D	c.308G > A	Missense	Tolerated(0.240)	p.P292S	c.874C > T	Missense	Damaging(0.000)
p.F121V	c.361T > G	Missense	Damaging(0.000)	p.D296H	c.886G > C	Missense	Damaging(0.000)
p.E127K	c.379G > A	Missense	Damaging(0.002)	p.Q304K	c.910C > A	Missense	Damaging(0.001)
p.E127G	c.380A > G	Missense	Damaging(0.001)	p.T328N	c.983C > A	Missense	Damaging(0.001)
p.G148R	c.442G > C	Missense	Damaging(0.000)	p.K335E	c.1003A > G	Missense	Damaging(0.026)
p.R169G	c.505C > G	Missense	Damaging(0.000)	p.Q429K	c.1285C > A	Missense	Tolerated(1.000)
p.R169S	c.505C > A	Missense	Damaging(0.001)	p.Y356D	c.1066T > G	Missense	Damaging(0.000)
p.H170P	c.509A > C	Missense	Damaging(0.001)	p.T372R	c.1115C > G	Missense	Damaging(0.000)
p.E178K	c.532G > A	Missense	Tolerated(0.054)	p.I406V	c.1216A > G	Missense	Damaging(0.03)
p.N223I	c.668A > T	Missense	Damaging(0.003)				

**Table 3:** The distribution of common PAH gene mutations within China.

Trivial name (protein effect)	Chinese mainland population								Taiwanese population
	Present study			p value	Previous study^12^			p value	Taiwanese study^40^
	RF(%)				RF(%)				RF(%)
	Chinese (n = 796)	Northern China (n = 557)	Southern China (n = 239)		Chinese (n = 212)	Northern China (n = 100)	Southern China (n = 112)		
p.R243Q	17.53	18.13	16.11	0.3300	25.43	25.50	23.21	0.6616	4.60
p.EX6-96A > G	7.66	7.54	7.95	0.7780	8.89	6.50	10.27	0.1463	9.20
p.V399V	5.84	6.64	3.97	0.0370**	3.21	2.50	3.57	0.4999	3.50
p.R241C	5.40	4.40	7.74	0.0070**	3.70	3.00	4.02	0.5453	23.20
p.R111*	4.77	5.39	3.35	0.0800	3.70	4.00	3.13	0.6536	3.50
p.Y356*	4.46	5.12	2.93	0.0530	5.19	5.55	4.46	0.6560	0.70
p.R413P	4.33	5.39	1.88	0.0020**	5.43	8.00	2.68	0.0155**	4.90
IVS4-1G > A	3.77	3.23	5.02	0.0860	6.42	6.00	6.25	0.8742	0.70

Abbreviation: *PAH*, phenylalanine hydroxylase; RF, relative frequency; **p < 0.05.

**Table 4:** Relative frequencies (%) of common *PAH* gene mutations detected in Asian and European populations.

Mutation	Asia					Europe			
	East Asia			West Asia		East Europe	West Europe	South Europe	North Europe
	Chinese n = 796	Japan^14^ n = 203	Korea^8^ n = 79	Turkish^18^ n = 569	Iran^17^ n = 124	Czech^28^ n = 665	German^5^ n = 226	Serbian^32^ n = 179	Dane^4^ n = 135
p.R243Q	17.53	5.40	12.00	NA	1.20	0.00	0.00	0.00	0.00
p.EX6-96A > G	7.66	4.70	10.10	NA	0.00	0.00	0.00	0.00	0.00
p.V399V	5.84	1.20	0.00	NA	0.00	0.00	0.00	0.00	0.00
p.R241C	5.40	10.10	5.70	NA	0.00	0.15	0.00	0.00	0.00
p.R111*	4.77	7.90	0.60	NA	0.00	0.00	0.00	2.60	0.30
p.Y356*	4.46	1.70	5.70	NA	0.00	0.00	0.91	0.00	0.30
p.R413P	4.33	24.10	3.20	NA	0.00	0.00	0.00	0.90	0.00
IVS4-1G > A	3.77	8.90	10.10	NA	0.00	0.00	0.00	0.00	0.00
p.A259T	0.00	1.00	5.70	NA	0.00	0.00	0.00	0.00	0.00
p.R252W	0.25	4.40	0.00	NA	4.80	3.83	0.46	0.00	0.00
p.T278I	0.19	4.20	3.20	NA	0.00	0.00	0.00	0.00	0.00
p.Y325*	0.57	0.00	3.20	NA	0.00	0.00	0.00	0.00	0.00
p.V388M	0.44	0.50	3.2	NA	0.00	0.15	0.00	0.00	0.30
p.L48S	0.06	0.00	0.00	7.00	0.80	1.88	0.02	31.00	0.00
IVS2 + 5G > C	0.19	0.00	0.00	NA	6.00	0.08	0.46	0.00	0.00
IVS4 + 5G > T	0.00	0.00	0.00	3.00	0.80	0.53	0.91	0.00	0.00
p.R243*	0.06	0.00	0.00	NA	6.90	2.71	2.05	1.70	0.00
p.R261Q	1.26	0.00	0.60	8.70	12.10	1.58	3.88	3.40	1.60
p.R261*	0.25	0.70	0.60	NA	4.80	0.08	0.00	1.70	0.00
p.P281L	0.19	0.00	1.90	8.40	8.50	2.03	3.65	6.00	1.30
p.A300S	0.00	0.00	0.00	5.00	0.40	0.98	0.46	0.00	0.00
IVS10-11G > A	0.25	0.00	0.00	24.60	24.60	3.61	2.97	1.70	5.20
p.K363Nfs*37	0.13	0.00	0.00	NA	7.30	0.00	0.00	0.00	0.00
p.E390G	0.00	0.00	0.00	4.10	0.00	0.23	0.91	5.20	0.00
IVS11 + 1G > C	0.00	0.00	0.00	NA	8.50	0.08	0.46	0.00	0.00
p.R408W	0.50	0.00	0.00	6.40	0.40	42.11	24.66	16.40	18.20
p.I65T	0.75	0.00	0.00	NA	0.00	1.28	0.00	0.00	0.70
p.R158Q	0.50	0.00	0.60	NA	0.00	4.14	4.57	3.40	2.90
p.R270K	0.00	0.00	0.00	NA	0.00	0.45	0.00	0.00	0.00
p.E280K	0.50	0.00	0.00	NA	0.80	0.00	0.00	0.00	2.90
p.I306V	0.00	0.00	0.00	NA	0.00	1.73	0.46	5.20	0.00
p.A403V	0.13	0.20	0.00	NA	0.00	5.11	5.71	1.70	0.00
p.Y414C	0.00	0.00	0.00	NA	0.00	1.58	4.57	1.70	10.10
IVS12 + 1G > A	0.00	0.00	0.00	NA	0.00	2.48	10.27	2.60	37.30

NA: not available.

**Table 5:** Patients’ phenotype and blood Phe levels.

patients’ phenotype	patients’ number	Phe levels (μmol/L)
		Mean ± SD
MHP	145	374.77 ± 141.29
mild PKU	215	919.22 ± 184.70
classic PKU	418	1872.55 ± 525.88
unclassified	18	not available
