# Supplementary material for: Molecular characterisation of phenylketonuria in a Chinese mainland population using next-generation sequencing
Source: Sci Rep. 2015 Oct 27;5:15769. doi: 10.1038/srep15769 (PMC4621502; doi:10.1038/srep15769)
Supplement: Supplementary Information [file srep15769-s1.pdf]

Molecular characterisation of phenylketonuria in a Chinese mainland population using next-generation sequencing

Nana Li[1,2,#], Haitao Jia[3,#], Zhen Liu[1,2], Jing Tao[1,2], Song Chen[3], Xiaohong Li[1,4], Ying Deng[1,2], Xi Jin[1,2,] Jiaping Song[3], Liangtao Zhang[3], Yu Liang[3], Wei Wang[3*], Jun Zhu[1,2,4*]

[1]National Center for Birth Defect Monitoring, West China Second University Hospital, Sichuan University, Sec.3 No.20, South RenMin Road, Chengdu, Sichuan, China

[2]Key Laboratory of Birth Defects and Related Diseases of Women and Children, Ministry of Education, West China Second University Hospital, Sichuan University, Sec.3 No.20, South RenMin Road, Chengdu, Sichuan, China

[3]BGI-Shenzhen, Building No.11, Beishan Industrial Zone, Yantian District, Shenzhen, Guangdong, China

[4]Laboratory of Molecular Epidemiology for birth defect, West China Institute of Women and Children's Health, Sichuan University, Chengdu, China

* Correspondence: zhujun028@163.com ,wangw@genomics.cn

[#]These authors contributed equally to this article

Supplementary Materials

Table S1. Patients' phenotype and blood Phe levels

No.	Phe levels (μmol/L)	Phenotype categories	No.	Phe levels (μmol/L)	Phenotype categories	No.	Phe levels (μmol/L)	Phenotype categories
1	126	MHP	261	936	mPKU	520	1620	cPKU
2	126	MHP	262	936	mPKU	521	1620	cPKU
3	127.8	MHP	263	938.4	mPKU	522	1620	cPKU
4	128.4	MHP	264	954	mPKU	523	1623	cPKU
5	133.8	MHP	265	954	mPKU	524	1629	cPKU
6	144	MHP	266	960	mPKU	525	1631.4	cPKU
7	147	MHP	267	960	mPKU	526	1632	cPKU
8	150	MHP	268	960	mPKU	527	1638	cPKU
9	156	MHP	269	963	mPKU	528	1639.2	cPKU
10	156.6	MHP	270	966	mPKU	529	1656	cPKU
11	162.6	MHP	271	966	mPKU	530	1656	cPKU
12	162.9	MHP	272	972	mPKU	531	1657.2	cPKU
13	168	MHP	273	984	mPKU	532	1662	cPKU
14	168	MHP	274	987	mPKU	533	1668.6	cPKU
15	168	MHP	275	1002	mPKU	534	1671	cPKU
16	172.2	MHP	276	1002	mPKU	535	1671	cPKU
17	174	MHP	277	1006.8	mPKU	536	1672.2	cPKU
18	180	MHP	278	1008	mPKU	537	1674	cPKU
19	180	MHP	279	1008	mPKU	538	1678.2	cPKU
20	180.6	MHP	280	1011.6	mPKU	539	1680	cPKU
21	181.8	MHP	281	1014	mPKU	540	1680	cPKU
22	183.78	MHP	282	1014	mPKU	541	1686	cPKU
23	186	MHP	283	1020	mPKU	542	1692	cPKU
24	190.8	MHP	284	1020	mPKU	543	1692	cPKU
25	197.4	MHP	285	1020	mPKU	544	1692	cPKU
26	197.4	MHP	286	1020	mPKU	545	1692	cPKU
27	198	MHP	287	1024.8	mPKU	546	1692.6	cPKU
28	201.6	MHP	288	1032	mPKU	547	1693.8	cPKU
29	204.6	MHP	289	1032	mPKU	548	1698	cPKU
30	207.6	MHP	290	1032	mPKU	549	1698	cPKU
31	213.6	MHP	291	1033.8	mPKU	550	1698	cPKU
32	225	MHP	292	1038	mPKU	551	1702.2	cPKU
33	225.6	MHP	293	1042.8	mPKU	552	1704	cPKU
34	232.2	MHP	294	1044	mPKU	553	1706.4	cPKU
35	240	MHP	295	1050	mPKU	554	1706.4	cPKU
36	252	MHP	296	1050	mPKU	555	1710	cPKU
37	252	MHP	297	1056	mPKU	556	1710	cPKU
38	253.8	MHP	298	1056	mPKU	557	1710	cPKU
39	255.6	MHP	299	1058.4	mPKU	558	1716	cPKU

Table 1 Continued

No.	Phe levels (μmol/L)	Phenotype categories	No.	Phe levels (μmol/L)	Phenotype categories	No.	Phe levels (μmol/L)	Phenotype categories
40	264	MHP	300	1062	mPKU	559	1716	cPKU
41	268.8	MHP	301	1062	mPKU	560	1727.4	cPKU
42	276	MHP	302	1062	mPKU	561	1734	cPKU
43	285.6	MHP	303	1068	mPKU	562	1739.1	cPKU
44	289.8	MHP	304	1068	mPKU	563	1740	cPKU
45	298.2	MHP	305	1074	mPKU	564	1746	cPKU
46	309	MHP	306	1080	mPKU	565	1749.6	cPKU
47	312	MHP	307	1080	mPKU	566	1758	cPKU
48	313.8	MHP	308	1080	mPKU	567	1758	cPKU
49	316.2	MHP	309	1080	mPKU	568	1770	cPKU
50	316.2	MHP	310	1086	mPKU	569	1770.54	cPKU
51	321.6	MHP	311	1091.4	mPKU	570	1773.6	cPKU
52	324	MHP	312	1104	mPKU	571	1775.4	cPKU
53	330	MHP	313	1104	mPKU	572	1776	cPKU
54	338.4	MHP	314	1110	mPKU	573	1776	cPKU
55	343.2	MHP	315	1115.4	mPKU	574	1776	cPKU
56	344.64	MHP	316	1116	mPKU	575	1777.8	cPKU
57	350.7	MHP	317	1122	mPKU	576	1779.6	cPKU
58	354	MHP	318	1122.6	mPKU	577	1779.6	cPKU
59	354	MHP	319	1126.2	mPKU	578	1782	cPKU
60	358.8	MHP	320	1134	mPKU	579	1782	cPKU
61	359.4	MHP	321	1135.2	mPKU	580	1782.6	cPKU
62	360	MHP	322	1137	mPKU	581	1782.6	cPKU
63	360	MHP	323	1140	mPKU	582	1788	cPKU
64	360	MHP	324	1140	mPKU	583	1788	cPKU
65	366	MHP	325	1140	mPKU	584	1794	cPKU
66	368.4	MHP	326	1140	mPKU	585	1800	cPKU
67	372	MHP	327	1140	mPKU	586	1800	cPKU
68	373.2	MHP	328	1143	mPKU	587	1800	cPKU
69	373.44	MHP	329	1146	mPKU	588	1800.9	cPKU
70	376.2	MHP	330	1146	mPKU	589	1806	cPKU
71	377.4	MHP	331	1152	mPKU	590	1812	cPKU
72	388.2	MHP	332	1153.2	mPKU	591	1812	cPKU
73	390	MHP	333	1164	mPKU	592	1812	cPKU
74	391.2	MHP	334	1170	mPKU	593	1818	cPKU
75	394.2	MHP	335	1170	mPKU	594	1830	cPKU
76	396	MHP	336	1170	mPKU	595	1830	cPKU
77	402	MHP	337	1171.8	mPKU	596	1830.6	cPKU
78	403.2	MHP	338	1176	mPKU	597	1833	cPKU
79	406.2	MHP	339	1176	mPKU	598	1836	cPKU
80	406.2	MHP	340	1180.2	mPKU	599	1836	cPKU

Table 1 Continued

No.	Phe levels (μmol/L)	Phenotype categories	No.	Phe levels (μmol/L)	Phenotype categories	No.	Phe levels (μmol/L)	Phenotype categories
81	408	MHP	341	1180.2	mPKU	600	1842	cPKU
82	412.2	MHP	342	1182	mPKU	601	1847.4	cPKU
83	414	MHP	343	1182	mPKU	602	1848	cPKU
84	419.4	MHP	344	1184.28	mPKU	603	1848.6	cPKU
85	420	MHP	345	1185	mPKU	604	1872	cPKU
86	420	MHP	346	1188	mPKU	605	1872	cPKU
87	420	MHP	347	1194	mPKU	606	1878	cPKU
88	421.8	MHP	348	1194	mPKU	607	1885.8	cPKU
89	426	MHP	349	1200	mPKU	608	1887	cPKU
90	428.4	MHP	350	1200	mPKU	609	1890	cPKU
91	432.6	MHP	351	1200	mPKU	610	1890	cPKU
92	444	MHP	352	1200	mPKU	611	1891.8	cPKU
93	444.6	MHP	353	1200	mPKU	612	1896	cPKU
94	450.6	MHP	354	1200	mPKU	613	1896	cPKU
95	454.8	MHP	355	1200	mPKU	614	1896.6	cPKU
96	456	MHP	356	1200	mPKU	615	1914	cPKU
97	456.6	MHP	357	1200	mPKU	616	1920	cPKU
98	459	MHP	358	1200	mPKU	617	1920.6	cPKU
99	462	MHP	359	1200	mPKU	618	1926	cPKU
100	467.4	MHP	360	1200	mPKU	619	1926	cPKU
101	474	MHP	361	1200.3	cPKU	620	1927.8	cPKU
102	474	MHP	362	1206	cPKU	621	1932	cPKU
103	474	MHP	363	1207.8	cPKU	622	1933.2	cPKU
104	479.4	MHP	364	1210.2	cPKU	623	1935	cPKU
105	480	MHP	365	1213.8	cPKU	624	1935.6	cPKU
106	480	MHP	366	1215	cPKU	625	1938	cPKU
107	480	MHP	367	1220.4	cPKU	626	1938	cPKU
108	480	MHP	368	1223.4	cPKU	627	1938	cPKU
109	488.4	MHP	369	1224	cPKU	628	1938	cPKU
110	499.8	MHP	370	1224	cPKU	629	1941	cPKU
111	499.8	MHP	371	1224	cPKU	630	1944	cPKU
112	500.4	MHP	372	1236	cPKU	631	1944	cPKU
113	502.02	MHP	373	1236	cPKU	632	1950	cPKU
114	507.6	MHP	374	1236.6	cPKU	633	1955.4	cPKU
115	510	MHP	375	1248	cPKU	634	1956	cPKU
116	512.4	MHP	376	1248	cPKU	635	1962	cPKU
117	522	MHP	377	1248	cPKU	636	1963.8	cPKU
118	522	MHP	378	1249.2	cPKU	637	1965	cPKU
119	534	MHP	379	1252.8	cPKU	638	1968	cPKU
120	534	MHP	380	1256.4	cPKU	639	1979.28	cPKU
121	534	MHP	381	1260	cPKU	640	1992	cPKU

Table 1 Continued

No.	Phe levels (μmol/L)	Phenotype categories	No.	Phe levels (μmol/L)	Phenotype categories	No.	Phe levels (μmol/L)	Phenotype categories
122	534	MHP	382	1260	cPKU	641	1997.4	cPKU
123	540	MHP	383	1260	cPKU	642	2010	cPKU
124	540	MHP	384	1260.6	cPKU	643	2010	cPKU
125	540	MHP	385	1260.6	cPKU	644	2016	cPKU
126	543.6	MHP	386	1265.4	cPKU	645	2016	cPKU
127	552	MHP	387	1266	cPKU	646	2020.2	cPKU
128	552	MHP	388	1266	cPKU	647	2022	cPKU
129	553.2	MHP	389	1272	cPKU	648	2022	cPKU
130	558	MHP	390	1278	cPKU	649	2040	cPKU
131	564	MHP	391	1284	cPKU	650	2042.4	cPKU
132	566.4	MHP	392	1298.4	cPKU	651	2051.4	cPKU
133	570	MHP	393	1299	cPKU	652	2052	cPKU
134	570	MHP	394	1302	cPKU	653	2058.6	cPKU
135	571.2	MHP	395	1302	cPKU	654	2064	cPKU
136	574.2	MHP	396	1302	cPKU	655	2066.4	cPKU
137	576.6	MHP	397	1308.6	cPKU	656	2066.4	cPKU
138	582	MHP	398	1320	cPKU	657	2070	cPKU
139	588.6	MHP	399	1320	cPKU	658	2070.6	cPKU
140	588.6	MHP	400	1320	cPKU	659	2082	cPKU
141	589.2	MHP	401	1326	cPKU	660	2083.56	cPKU
142	591.6	MHP	402	1326	cPKU	661	2093.76	cPKU
143	592.2	MHP	403	1326	cPKU	662	2094	cPKU
144	597	MHP	404	1326	cPKU	663	2094	cPKU
145	598.8	MHP	405	1332	cPKU	664	2104.44	cPKU
146	600	mPKU	406	1332	cPKU	665	2108.4	cPKU
147	604.8	mPKU	407	1332	cPKU	666	2115	cPKU
148	605.4	mPKU	408	1348.8	cPKU	667	2118	cPKU
149	606	mPKU	409	1350	cPKU	668	2124	cPKU
150	611.4	mPKU	410	1350	cPKU	669	2136	cPKU
151	616.8	mPKU	411	1355.88	cPKU	670	2142	cPKU
152	618	mPKU	412	1356	cPKU	671	2143.2	cPKU
153	618	mPKU	413	1356	cPKU	672	2154	cPKU
154	618	mPKU	414	1356	cPKU	673	2154.6	cPKU
155	619.2	mPKU	415	1359.6	cPKU	674	2156.4	cPKU
156	624	mPKU	416	1360.2	cPKU	675	2174.4	cPKU
157	630	mPKU	417	1360.32	cPKU	676	2179.2	cPKU
158	630	mPKU	418	1362	cPKU	677	2191.56	cPKU
159	631.8	mPKU	419	1368	cPKU	678	2208	cPKU
160	636	mPKU	420	1374	cPKU	679	2211	cPKU
161	636	mPKU	421	1378.2	cPKU	680	2214	cPKU
162	642	mPKU	422	1380	cPKU	681	2214	cPKU

Table 1 Continued

No.	Phe levels (μmol/L)	Phenotype categories	No.	Phe levels (μmol/L)	Phenotype categories	No.	Phe levels (μmol/L)	Phenotype categories
163	642.6	mPKU	423	1380	cPKU	682	2214	cPKU
164	651	mPKU	424	1386	cPKU	683	2216.4	cPKU
165	654	mPKU	425	1386	cPKU	684	2220	cPKU
166	657.6	mPKU	426	1392	cPKU	685	2227.8	cPKU
167	660	mPKU	427	1392	cPKU	686	2232	cPKU
168	660.6	mPKU	428	1392	cPKU	687	2236.2	cPKU
169	665.4	mPKU	429	1392	cPKU	688	2236.8	cPKU
170	666.6	mPKU	430	1393.2	cPKU	689	2237.52	cPKU
171	672	mPKU	431	1398	cPKU	690	2238	cPKU
172	672	mPKU	432	1398	cPKU	691	2244	cPKU
173	672	mPKU	433	1400.4	cPKU	692	2250.6	cPKU
174	678.6	mPKU	434	1413.6	cPKU	693	2256	cPKU
175	685.2	mPKU	435	1416	cPKU	694	2256	cPKU
176	699.6	mPKU	436	1416	cPKU	695	2256	cPKU
177	700.8	mPKU	437	1418.4	cPKU	696	2259	cPKU
178	702	mPKU	438	1420.8	cPKU	697	2262	cPKU
179	705	mPKU	439	1422	cPKU	698	2274	cPKU
180	706.8	mPKU	440	1422	cPKU	699	2280	cPKU
181	708	mPKU	441	1422	cPKU	700	2294.4	cPKU
182	708	mPKU	442	1428	cPKU	701	2298	cPKU
183	709.8	mPKU	443	1434	cPKU	702	2298	cPKU
184	714	mPKU	444	1434	cPKU	703	2298	cPKU
185	718.8	mPKU	445	1434	cPKU	704	2316	cPKU
186	720	mPKU	446	1434	cPKU	705	2318.4	cPKU
187	720	mPKU	447	1434	cPKU	706	2319	cPKU
188	720	mPKU	448	1440	cPKU	707	2328	cPKU
189	726	mPKU	449	1440	cPKU	708	2334	cPKU
190	726	mPKU	450	1441.8	cPKU	709	2337	cPKU
191	726	mPKU	451	1446	cPKU	710	2340	cPKU
192	732	mPKU	452	1446	cPKU	711	2363.4	cPKU
193	732.6	mPKU	453	1451.7	cPKU	712	2368.8	cPKU
194	737.1	mPKU	454	1452	cPKU	713	2370	cPKU
195	738	mPKU	455	1452	cPKU	714	2370.6	cPKU
196	738	mPKU	456	1458	cPKU	715	2371.8	cPKU
197	738	mPKU	457	1458	cPKU	716	2400	cPKU
198	750	mPKU	458	1458	cPKU	717	2400	cPKU
199	757.2	mPKU	459	1458	cPKU	718	2402.4	cPKU
200	761.4	mPKU	460	1458	cPKU	719	2412	cPKU
201	768	mPKU	461	1458	cPKU	720	2424	cPKU
202	780	mPKU	462	1464	cPKU	721	2430	cPKU
203	780	mPKU	463	1464.6	cPKU	722	2430	cPKU

Table 1 Continued

No.	Phe levels (μmol/L)	Phenotype categories	No.	Phe levels (μmol/L)	Phenotype categories	No.	Phe levels (μmol/L)	Phenotype categories
204	780	mPKU	464	1470	cPKU	723	2434.5	cPKU
205	789.6	mPKU	465	1470	cPKU	724	2437.8	cPKU
206	790.8	mPKU	466	1482	cPKU	725	2442	cPKU
207	792	mPKU	467	1482	cPKU	726	2442	cPKU
208	801	mPKU	468	1482	cPKU	727	2448	cPKU
209	804	mPKU	469	1482	cPKU	728	2449.74	cPKU
210	804.6	mPKU	470	1488	cPKU	729	2460	cPKU
211	811.8	mPKU	471	1488	cPKU	730	2472.54	cPKU
212	814.2	mPKU	472	1488	cPKU	731	2478	cPKU
213	815.4	mPKU	473	1494	cPKU	732	2514	cPKU
214	816	mPKU	474	1500	cPKU	733	2514	cPKU
215	817.8	mPKU	475	1500	cPKU	734	2515.2	cPKU
216	817.8	mPKU	476	1500	cPKU	735	2516.4	cPKU
217	821.4	mPKU	477	1500	cPKU	736	2520	cPKU
218	822	mPKU	478	1503	cPKU	737	2520	cPKU
219	828	mPKU	479	1504.8	cPKU	738	2520	cPKU
220	828	mPKU	480	1512	cPKU	739	2533.8	cPKU
221	834	mPKU	481	1512	cPKU	740	2545.8	cPKU
222	834	mPKU	482	1519.8	cPKU	741	2556	cPKU
223	834.18	mPKU	483	1524.6	cPKU	742	2596.8	cPKU
224	835.2	mPKU	484	1527	cPKU	743	2604	cPKU
225	840	mPKU	485	1530	cPKU	744	2611.8	cPKU
226	840	mPKU	486	1530	cPKU	745	2627.4	cPKU
227	840	mPKU	487	1530	cPKU	746	2628	cPKU
228	843.6	mPKU	488	1536	cPKU	747	2634	cPKU
229	846	mPKU	489	1542	cPKU	748	2634	cPKU
230	848.4	mPKU	490	1542	cPKU	749	2660.4	cPKU
231	849.06	mPKU	491	1548	cPKU	750	2680.2	cPKU
232	852	mPKU	492	1548	cPKU	751	2688	cPKU
233	855.6	mPKU	493	1548	cPKU	752	2700	cPKU
234	864	mPKU	494	1557	cPKU	753	2700	cPKU
235	864	mPKU	495	1558.2	cPKU	754	2712	cPKU
236	864	mPKU	496	1561.8	cPKU	755	2741.4	cPKU
237	864	mPKU	497	1568.28	cPKU	756	2784	cPKU
238	864	mPKU	498	1572	cPKU	757	2815.8	cPKU
239	882	mPKU	499	1572	cPKU	758	2824.2	cPKU
240	882	mPKU	500	1574.4	cPKU	759	2856	cPKU
241	885	mPKU	501	1576.2	cPKU	760	2884.8	cPKU
242	885.6	mPKU	502	1578	cPKU	761	2940	cPKU
243	889.2	mPKU	503	1578	cPKU	762	2970	cPKU
244	900	mPKU	504	1578	cPKU	763	2974.2	cPKU

Table 1 Continued

No.	Phe levels (μmol/L)	Phenotype categories	No.	Phe levels (μmol/L)	Phenotype categories	No.	Phe levels (μmol/L)	Phenotype categories
245	900	mPKU	505	1584	cPKU	764	2998.5	cPKU
246	900	mPKU	506	1584	cPKU	765	3030	cPKU
247	900	mPKU	507	1590	cPKU	766	3036	cPKU
248	900	mPKU	508	1594.8	cPKU	767	3107.4	cPKU
249	903.6	mPKU	509	1596	cPKU	768	3129.6	cPKU
250	908.4	mPKU	510	1596	cPKU	769	3162	cPKU
251	912	mPKU	511	1599	cPKU	770	3329.34	cPKU
252	912	mPKU	512	1599	cPKU	771	3464.7	cPKU
253	914.4	mPKU	513	1600.8	cPKU	772	3468	cPKU
254	918	mPKU	514	1602	cPKU	773	3479.4	cPKU
255	918	mPKU	515	1602.6	cPKU	774	3487.2	cPKU
256	919.2	mPKU	516	1608	cPKU	775	3613.2	cPKU
257	924	mPKU	517	1613.4	cPKU	776	3780	cPKU
258	924	mPKU	518	1614	cPKU	777	4080	cPKU
259	926.4	mPKU	519	1618.2	cPKU	778	5160	cPKU
260	933	mPKU						

Abbreviations:

Phe: phenylalanine

MHP: mild hyperphenylalaninemia

mPKU: mild PKU

cPKU: classic PKU

*The pretreatment Phe levels of the remaining 18 patients were not available, their phenotypes could not be classified.
